# Utilizing Co-Creative Principles to Develop an E-Learning Platform for Interprofessional Training on Tinnitus: The Erasmus+ Project Tin-TRAC

**DOI:** 10.3390/ijerph19148323

**Published:** 2022-07-07

**Authors:** Evangelos Paraskevopoulos, Marios Avraamides, Panagiotis D. Bamidis, Christian Dobel, Sotiria Gilou, Christos I. Ioannou, Dimitris Kikidis, Birgit Mazurek, Winfried Schlee, Andria Shimi, Eleftheria Vellidou

**Affiliations:** 1 Department of Psychology, University of Cyprus, Nicosia 1678, Cyprus; mariosav@ucy.ac.cy (M.A.); shimi.andria@ucy.ac.cy (A.S.); 2 Laboratory of Medical Physics and Digital Innovation, School of Medicine, Faculty of Health Sciences, Aristotle University of Thessaloniki, 54124 Thessaloniki, Greece; bamidis@med.auth.gr (P.D.B.); sotiriagilou@gmail.com (S.G.); 3 CYENS—Centre of Excellence, Nicosia 1016, Cyprus; christos.ioannou@cyens.org.cy; 4 Department of Otorhinolaryngology, Jena University Hospital, Friedrich Schiller University, 07743 Jena, Germany; christian.dobel@med.uni-jena.de; 5 Department of Otolaryngology, Hippocrateion Hospital, National and Kapodistrian University of Athens, 15772 Athens, Greece; dimitriskikidis@yahoo.com; 6 Tinnitus Center, Charité—Universitätsmedizin Berlin, 10117 Berlin, Germany; birgit.mazurek@charite.de; 7 Department of Psychiatry and Psychotherapie, University of Regensburg, 93053 Regensburg, Germany; winfried.schlee@gmail.com; 8 Institute of Communication and Computer Systems (ICCS), 10682 Athens, Greece; ebel@biomed.ntua.gr

**Keywords:** tinnitus, interprofessional training, reusable learning objects, co-creation

## Abstract

Tinnitus treatment, diagnosis and management across Europe varies significantly. The lack of national clinical guidelines for tinnitus management in most European countries and the absence of a common language across all disciplines involved is reflected in the diversification of healthcare practices. Interprofessional Training for Tinnitus Researchers and Clinicians (Tin-TRAC) is an Erasmus+ project that aims to develop common educational ground in the form of an e-Learning platform, co-created by patients, researchers and clinicians, which is able to unify tinnitus diagnosis and treatment strategies across Europe. A pan-European thematic educational platform integrating the best practices and latest research achievements with regard to tinnitus diagnosis and management has the potential to act as a facilitator of the reduction of interdisciplinary and interregional practice diversification. A detailed analysis of the educational needs of clinicians and researchers across disciplines will be followed by the co-creative development of the curriculum. Reusable learning objects will incorporate the training contents and will be integrated in an open e-Learning platform. Tin-TRAC envisions that its output will answer the need to create a common language across the clinicians and researchers of different disciplines that are involved in tinnitus management, and reduce patients’ prolonged suffering, non-adherence and endless referral trajectories.

## 1. Introduction

Tinnitus is defined as the sensation of noise in the absence of a corresponding external sound. It consists of two parameters: a phantom ringing, hissing or buzzing in the ears or head, and the degree of emotional reaction to this percept [1]. Chronic tinnitus is related to significant distress and reduction in quality of life, and is accompanied by sleeping problems, stress, depression and substance abuse [2]. Epidemiological studies suggest that it affects 10–30% of the adult population in EU countries, while its incidence reaches 5.4 new cases/10,000 inhabitants [3], and approximately 65 million adults in EU28 have tinnitus [4]. In addition to the societal and individual burden, the economic one is vast as well. For each of the affected patients, the cost of tinnitus healthcare is estimated to be EUR 500–1500 per year [5].

As the most prominent elements of tinnitus are not measurable or quantifiable by objective physical recordings, understanding of its causes, as well as patients’ heterogeneity, is obstructed. Indeed, the great variability of patient characteristics, which include differences in etiology, geno- and phenotyping, is still poorly understood. The current working model of tinnitus pathophysiology [6] does not fully explain its chronification, nor the discomfort it causes [7]. This, in turn, justifies the minimal impact that these recent scientific advances have in clinical practice [8]. However, this seems to affect patients’ treatment quality. Recent evidence suggests that tinnitus diagnosis is often a subject of prolonged referral trajectories, resulting in an amplification of the corresponding psychological and societal burden [9].

A recent study on the variation in clinicians’ and stakeholders’ opinions and practices regarding tinnitus across 24 European countries revealed significant differences in healthcare structures and practices followed [10], especially with regard to its cognitive dimension. Interdisciplinary approach and individualized treatment plans are key points for the successful management of tinnitus, due to the great heterogeneity of symptoms. ENT physicians, audiologists, psychiatrists, psychologists, neurologists, behavioral neuroscientists, general practitioners, nurses, psychoacousticians, basic scientists and patient representatives may collaborate to provide highly personalized and up-to-date diagnostic and management services focusing on tinnitus pathophysiology, assessment and possible cures. Nonetheless, the lack of national clinical guidelines for the management of tinnitus in most European countries [11] and the absence of a common language across all disciplines involved is reflected in this diversification of healthcare practices, which acts as a barrier in tinnitus management progress.

## 2. Towards a Co-Creatively Developed Interprofessional Training for Tinnitus

A pan-European thematic educational platform integrating the best practices and latest research achievements with regard to tinnitus diagnosis and management, highlighting its cognitive dimension, has the potential to act as a facilitator to the reduction of this diversification. 

Interprofessional Training for Tinnitus Researchers and Clinicians (Tin-TRAC) is an Erasmus+ project, funded as a cooperation partnership in vocational education and training under the Grant Agreement number 2021-1-CY01-KA220-VET-000025455. The project was officially started on 25 February 2022 and comprises six educational, research and clinical centers in three European countries, namely Cyprus, Germany and Greece. Tin-TRAC’s administration is located in Cyprus, where it is managed by Project Coordinator Evangelos Paraskevopoulos at the Department of Psychology in the University of Cyprus. More information about the Tin-TRAC and the project’s process can be followed on its website https://tin-trac.eu, accessed on 4 July 2022.

Tin-TRAC envisions the creation of a pan-European network whose collaboration will focus on educating professionals from varied backgrounds via a horizontal thematic approach to exploit recent (neuro)scientific advances in the field and acquire tinnitus specialized skills through webinars, workshops, scientific exchange and innovative learning objectives. It will identify the best practices, policies and methods regarding specialized training across Europe and organize thematic training programs for researchers and clinicians throughout different European facilities. Co-creation principles will be utilized to develop an e-Learning platform with reusable learning resources understandable for the wide range of healthcare providers and researchers involved in tinnitus management.

The long-term ambition of Tin-TRAC is to develop a common educational ground, co-created by patients, researchers and clinicians of different disciplines, to unify the diagnosis and treatment strategies followed in tinnitus across Europe. This will answer the need to create common language and thereby reduce patients’ increasing complaints, prolonged suffering, non-adherence, healthcare overuse and endless referral trajectories. A consensus on assessment and treatment will foster treatment stability and care provision. At the same time, the content of the learning objects will be utilized by the standard curriculum of the educational and research institutions, answering the need to use high-quality innovative learning objects and strategies to teach healthcare students and train the trainers and researchers via methodologies that are in line with learners’ needs and objectives. Table 1 depicts the main objectives of Tin-TRAC. 

## 3. Concept and Methodology

Tin-TRAC will promote the inter-connection of higher education systems of Europe via the formation of a network across different European universities and clinics, where work-based knowledge and methodology will be transferred. A specific curriculum regarding training activities that will guide the training of researchers and clinicians via an e-Learning platform will be one of the major contributions of the project to the European and international community of tinnitus experts. Innovative methods and pedagogies based on co-creative elements [12] will draw on best practices from clinicians, researchers and trainers exploring the development of collective practices in research and clinical settings, allowing the development of novel collaborations in terms of teaching and learning, as well as in terms of research.

At the preparation stage, Tin-TRAC designed 6 project results, gradually developing the overall goal of a pan-European educational platform for interprofessional training for tinnitus. These will implement: (1) a needs analysis regarding the educational inconsistencies and variabilities amongst researchers and practitioners of different disciplines with regard to tinnitus diagnosis and management; (2) a training curriculum including the contents of the educational goals, as informed by the needs analysis; (3) the development of Reusable Learning Objects as agile methods for emergent learning resources [13]; (4) the development and deployment of the e-Learning platform that will host the RLOs; (5) the evaluation of project outcomes; (6) and the generation of a best practices and recommendations report. These project results will emerge from learning and teaching activities following a framework of active involvement of researchers and clinicians, collectively shaping and co-creating the content of the education. These activities will be held across all 3 countries with varying contributions among the consortium.

### 3.1. Needs Analysis

The first project result (PR) has a twofold purpose: (a) to identify the patients’ needs with regard to healthcare and (b) to identify the educational needs and interdisciplinary inconsistencies in tinnitus researchers and clinicians. In particular, it will investigate key clinical areas where the educational resources, including the RLOs, have to focus, and the information that should be included. Building on prior studies that identified inconsistencies in clinical practice [10], this activity will enhance our understanding of the educational needs and inconsistencies across disciplines. A questionnaire focusing on educational needs will be disseminated in the target population of each partner’s country and the results will be analyzed via descriptive methodology. Thereby, it will listen to the target population and provide raw data about the differences between the typical healthcare practice with regard to tinnitus in the EU.

To this extent, data will enable the integration of clinical and research practice, fostering effective communication between members of interdisciplinary teams dealing with tinnitus through Tin-TRAC’s creative digital resources. Hence, the target population will shape the content of Tin-TRAC’s output, allowing the development of state-of-the-art educational content that answers the corresponding needs effectively. This will inform the educational material to be developed in the following project results.

### 3.2. Training Curriculum

The second project result aims to design and develop the Continuing Vocational Education and Training Curriculum based on the analysis. The curriculum will refer to the knowledge and skills trainees are expected to acquire, which includes the training objectives they are expected to meet, the subject matters covered and the materials available. The goal of this PR is to compile a curriculum fully specialized and targeted to the needs of the trainees, as identified from the corresponding needs analysis. The learning objectives (information to be learned and applied by the patients) will be prioritized in a standardized way that follows a common syllabus, but at the same time allows learning resources to function independently [14]. A leveled presentation of the final curriculum will allow the material to incorporate the educational needs not only of the clinicians and researchers, but also of the patients, who need to know more about their condition in order to participate actively in its management.

A learning outcomes approach will be used, based on the principles of the European Credit System for Vocational Education and Training (ECVET) [15] to enable the development of a Sectoral Qualifications Framework within the framework of European Qualifications Framework (EQF) and future recognition and accreditation. All partners will be involved in the development of the curriculum. The main tasks of the curriculum will be to (a) co-create the content with the involved stakeholders to establish the curriculum framework and (b) develop the curriculum.

The material will be disseminated through the e-Learning platform, international conferences and international journals. It will be integrated to the standard curriculum of the educational and research institutions, answering the need to use high-quality innovative learning objects and strategies to teach healthcare students and train the trainers and researchers via methodologies that are in line with learners’ needs and objectives.

### 3.3. Co-Creation Principles in Shaping Learning Objectives

Co-creation strategies allow educators and learners to work together to improve the learners’ experience and enhance students’ ability to act as partners [12]. In such approaches, learners’ efforts are integrated with educators’ resources to facilitate actions and practices that motivate exchange and communication, leading to better educational experiences implemented by innovative pedagogical methods [16]. Iterative activities that allow the listening of the educational needs of tinnitus researchers, clinicians and patients (PR 1) and the shaping of the educational content (learning and teaching activities that foster the development of the curriculum) will ensure the active engagement of the target population throughout the project’s lifetime. Prior work on incorporating patients’ stories, such as the platform Siopi (https://siopi.ai/contribute-lp, accessed on 4 July 2022), may provide interesting data on patients’ views.

### 3.4. Development of Reusable Learning Objects

The creative digital resources design aims to keep the control of the process in the hands of the content experts. The ASPIRE framework will be used which stands for the stages of the process—A—Aims, S—Storyboard, P—Population and Production, I—Integration, R—Release and E—Evaluate [17]. This framework will allow the information to be storyboarded and populate it in a creative interactive multimedia resources platform. Building upon granular principles and highly focused on a single learning goal, such learning resources are typically interactive, visual, small in size and highly aligned with their perceived learning needs. The form of the RLOs developed will permit repurposing towards the coverage of a variety of educational needs of different curricula, thus allowing their integration to standard courses of the educational centers involved in Tin-TRAC [18].

### 3.5. Development and Deployment of the E-Learning Platform

Following the definition of the curriculum contents and the creation of the RLOs, an e-Learning platform will be developed and deployed. The platform’s requirements will be elicited through a checklist while the content will be co-created by experts of all partners. The RLOs will be unified within a Massive Online Open Course (MOOC), which will be open and available for everyone and especially for tinnitus patients, in order to acquire practical knowledge about the differences of tinnitus evaluation and management in the different countries from the clinicians and researchers of different disciplines. In order to ensure the maximum quality of Tin-TRAC’s course, the partners will follow the Design and Quality of Online Courses checklist [19]. These requirements include the following categories: analysis, design, implementation, realization and evaluation. In particular, the platform will include: subject-specific video lectures, 6–9 min long, with embedded self-assessment questionnaires; teaching and learning activities including multiple choice questions (MCQs); and case-based formative, learning-focused assessment for self-study. Finally, the platform will include a forum where the participants can interact.

Tin-TRAC envisions that the MOOC will maximize the impact of the created educational material and will increase the possibilities of integration of practices between researchers and clinicians.

## 4. Evaluation of Project Outcomes and Generation of a Best Practices and Recommendations Report

The two final goals of Tin-TRAC are to evaluate the project outcomes and create a best practices and recommendations report referring to the methodology of co-creatively developing interdisciplinary thematic MOOCs. The data generated by this intellectual output will offer material for dissemination and encourage further initiatives. Dissemination of this result to stakeholders, universities, clinics, researchers and practitioners will enable the exploitation of e-Learning resources developed, as well as the standardization of the corresponding methodologies. The goals of these PRs will be achieved via activities implementing dissemination of questionnaires that will be based on previous partners’ experience and will aim to evaluate the project’s success. A researcher with expertise in qualitative research will format the semi-structured interviews and will manage the overall organization. Data will be triangulated from different sources in order to obtain a more complete picture via: (a) user stories (focus group, personal interview); (b) satisfactory surveys (online and offline surveys); (c) complaints (personal interview, complaints forms); and (d) general feedback. A Net Promoter Score will be used both to measure the performance and to increase dissemination. This will measure the likeliness of a user referring Tin-TRAC resources to someone else, showing commitment and satisfaction. Descriptive statistics will be used to provide evidence about the effectiveness and acceptance of the digital resources and the MOOC course developed.

## 5. Conclusions

Overall, this PR will provide evidence about the effectiveness of the project and therefore encourage the development of further educational advancements targeting tinnitus evaluation and management. At the same time, it will provide a roadmap regarding the implementation of such a project for different healthcare conditions.

## Figures and Tables

**Table 1:** Main objectives of Tin-TRAC.

Provide a pan-European framework for interdisciplinary exchange of knowledge and cooperation.
Identify the best practices, policies and methods regarding specialized training across Europe and organize customized training programs for researchers and clinicians throughout different European facilities.
Offer the possibility of collaboration with organizations in other sectors in order to stimulate a more comprehensive, rapid, effective and long-term response to tinnitus patients.
Exploit co-creative practices on the preparation and dissemination of comprehensive high-quality educational material on globally accepted guidelines on tinnitus assessment and management.
Train possible future trainers of health care professionals. Multiply impact, create long-term effect.

## Data Availability

Not applicable.
